# First- vs. Second-Generation Autologous Platelet Concentrates and Their Implications for Wound Healing: Differences in Proteome and Secretome

**DOI:** 10.3390/bioengineering11111171

**Published:** 2024-11-20

**Authors:** Hanna L. Stiller, Natarajan Perumal, Caroline Manicam, Emily R. Trzeciak, Julia Todt, Kerstin Jurk, Andrea Tuettenberg, Sven Schumann, Eik Schiegnitz, Sebastian Blatt

**Affiliations:** 1 Department of Oral and Maxillofacial Surgery, University Medical Center, Johannes Gutenberg-University Mainz, 55131 Mainz, Germany; hanna.stiller@unimedizin-mainz.de (H.L.S.); jtodt@uni-mainz.de (J.T.); eik.schiegnitz@unimedizin-mainz.de (E.S.); 2 Department of Ophthalmology, University Medical Center, Johannes Gutenberg-University Mainz, 55131 Mainz, Germanycaroline.manicam@unimedizin-mainz.de (C.M.); 3 Department of Dermatology, University Medical Center, Johannes Gutenberg-University Mainz, 55131 Mainz, Germany; etrzecia@students.uni-mainz.de (E.R.T.); antuette@uni-mainz.de (A.T.); 4 Center for Thrombosis and Hemostasis, University Medical Center, Johannes Gutenberg-University Mainz, 55131 Mainz, Germany; kerstin.jurk@unimedizin-mainz.de; 5 Research Center for Immunotherapy, University Medical Center, Johannes Gutenberg-University Mainz, 55131 Mainz, Germany; 6 Institute of Anatomy, Brandenburg Medical School Theodor Fontane, 16816 Neuruppin, Germany; sven.schumann@mhb-fontane.de; 7 Platform for Biomaterial Research, BiomaTiCS Group, University Medical Center, Johannes Gutenberg-University Mainz, 55131 Mainz, Germany

**Keywords:** platelet-rich plasma, platelet-rich fibrin, reconstruction, angiogenesis, vascularization, proteomics

## Abstract

Differences in cell count and growth factor expression between first- and second-generation autologous platelet concentrates (APCs) have been well described. The debate over which formula best supports wound healing in various surgical procedures is still ongoing. This study aims to assess the whole proteome assembly, cell content, immunological potential and pro-angiogenic potential of second-generation APC, Platelet-Rich Fibrin (PRF) vs. first-generation APC, Platelet-Rich Plasma (PRP). The global proteome of the APCs was analyzed using nano-liquid chromatography mass spectrometry. Blood cell concentrations were determined by an automated cell counter. The effect of APCs on macrophage polarization was analyzed by flow cytometry. A yolk sac membrane (YSM) assay was used to monitor the neo-vessel formation and capillary branching in vivo. Cell count analysis revealed a higher number/concentration of leukocytes in PRF vs. PRP. Incubation of macrophages with PRP or platelet-free plasma (PFP) did not induce a significant pro-inflammatory state but led to a shift to the M0/M2 phenotype as seen in wound healing for all tested formulas. Label-free proteomics analysis identified a total of 387 proteins from three biological replicates of the respective designated groups. PRF induced increased formation of neo-vessels and branching points in vivo in comparison to PRP and PFP (each *p* < 0.001), indicating the enhanced pro-angiogenic potential of PRF. Overall, PRF seems superior to PRP, an important representative of first-generation formulas. Inclusion of leucocytes in PRF compared to PRP suggested rather an anti-inflammatory effect on macrophages. These results are important to support the versatile clinical applications in regenerative medicine for second-generation autologous platelet concentrates to optimize wound healing.

## 1. Introduction

Autologous platelet concentrates (APCs) were developed as an advanced version of the fibrin sealants more than 40 years ago [1]. Traditionally known for their roles in hemostasis and thrombosis, platelets have also emerged as crucial mediators of many other pathophysiological processes including tumor metastasis, autoimmune diseases, and neurodegenerative diseases. Platelets regulate the innate and adaptive immunity, angiogenesis, and vessel formation and can ultimately be seen as an innovative drug delivery system, useful in regenerative medicine [2]. Therefore, APCs are now considered essential in various surgical fields [3]. Clinical indications for APCs are versatile in oral and maxillofacial surgery including but not limited to periodontal and dentoalveolar surgery, bone regeneration, implantology, and reconstructive surgery [4]. To define the wide range in formulas, centrifugation processes, and production techniques that results in different products with different biological activity and potential use, a classification protocol for APCs was established [5]. Four main groups of APCs are classified, depending on their leukocyte and fibrin content, comprising pure platelet-rich plasma (P-PRP), leukocyte- and platelet-rich plasma (L-PRP), pure platelet-rich fibrin (P-PRF), and leukocyte- and platelet-rich fibrin (L-PRF) [5]. However, emerging and underlying commercial interests applied altered centrifugation protocols, leading to new subclassifications and the comparability of scientific evidence is hampered [3]. As first-generation APCs, PRP with its different formulas has been extensively studied in regenerative therapies. However, the major drawbacks of this technique are non-standardized isolation protocols, a multi-step centrifugation process that involves the addition of anticoagulants, and the rapid outburst of growth factors that can lead to a growth factor release after activation of 95% within 10 min [6]. To overcome these limitations, second-generation APCs such as PRF were developed [4]. They are manufactured in a one-step centrifugation process without the addition of anticoagulants and other substances. Of note, the flexible fibrin mesh can act as a scaffold for entrapped blood cells to increase the angiogenic, osteogenic, and antimicrobial potential in tissue regeneration. Here, PRF is described as a biodegradable biopolymer by creating a three-dimensional structure with enclosed platelet-derived growth factors, thus facilitating cell adhesion and proliferation [3]. This allows the slow and steady release of growth factors such as the Vascular Endothelial Growth Factor (VEGF) that is in accordance with physiological release kinetics in wound-healing processes [7].

Multiple preclinical studies have documented differences in cell counts, particularly leukocytes, and the growth factor expression between first- and second-generation formulas. Recent evidence favors PRF for long-term release at a generally higher level of growth factor content [8,9,10]. However, especially the enriched leukocyte number is considered particularly critical as it was linked to an increased pro-inflammatory potential [7]. Generally, the discussion on which formula is better suited in maxillofacial tissue regeneration procedures is still ongoing [5,10].

As a general problem, evidence is often based on in vitro data, which are hampered by non-standardized protocols and missing information for APC production [11]. Furthermore, the proteome landscape of the different sub-classes of APC is largely unknown [12,13]. Few studies, such as Al-Sharabi et al., focusing on the secretome of the respective APCs reported over 700 proteins representing not only growth factors but also various cytokine- and immune-related proteins [13]. So far, the direct comparison between PRF and PRP remains to be explored.

This study aimed to evaluate the differences in blood cell count, overall proteome composition, immunological implications like pro- or anti-inflammatory effects, and the pro-angiogenic potential in vivo of first-generation APCs (PRP) and second-generation APCs (PRF).

## 2. Materials and Methods

### 2.1. Preparation of APC

#### 2.1.1. Generation of Platelet-Rich Fibrin (PRF)

All APCs used in this study were prepared from the peripheral venous blood of four healthy volunteers without any systemic health issues and intake of platelet function affecting medication for at least 10 days as well as no history of smoking. Healthy, non-smoking donors without any systemic health issue were utilized to minimize variability in the cell function, ensuring consistency in the comparative analysis of APCs. All procedures were conducted in accordance with the Declaration of Helsinki and approved by the Ethics Committee of the Landesärztekammer Rhineland-Palatine (no. 2019-14705_1, 2023-17021). PRF was generated as previously described [14]. In brief, after a venous puncture, 10 mL of blood was collected from every donor via a special vacutainer system (A-PRF+; Process for PRF, Nice, France). After immediate centrifugation at 1200 rpm for 8 min (177× *g* at a fixed angle rotor with 110 mm radius, Duo centrifuge, Mectron, Carasco, Italy), a liquid and clotted phase of PRF could be obtained. The centrifugation protocols were chosen based on their widespread preclinical and clinical use in previously conducted studies [14].

#### 2.1.2. Isolation of Platelet-Rich Plasma (PRP)

Platelet-Rich Plasma (PRP) was prepared as previously published [15]. Briefly, a venous blood sample of 3 mL was drawn, anticoagulated with trisodium citrate (0.0108 M/L), and processed within 1 h of collection. Differential centrifugation (200× *g* for 10 min at room temperature, Thermo Fisher Scientific, Heraeus Megafuge 16) was performed to generate PRP. To obtain clotted PRP, the sample was incubated with 25 mM CaCl_2_, vortexed, and incubated at 37 °C in a water bath for 30 min followed by a frozen cycle at −20 °C and subsequent centrifugation at 2000× *g* for 10 min at room temperature.

#### 2.1.3. Preparation of Platelet-Free Plasma (PFP)

For control experiments, platelet-free plasma (PFP) was manufactured adopted to a method previously described [16]. Platelet-rich plasma (PRP) and platelet-poor plasma (PPP) were prepared by a centrifugation of citrated whole blood as described [15]. Platelet-free plasma (PFP) was prepared by a centrifugation of PPP (30,000× *g* for 10 min at room temperature, Thermo Fisher Scientific, Waltham, MA, USA, Fresco 21) and incubated with the snake venom batroxobin (Loxo, Dossenheim, Germany; a final concentration 0.25 U/mL) for 30 min at 37 °C in a water bath to form clotted PFP. This was followed by a frozen cycle at −20 °C and subsequent centrifugation at 2000× *g* for 10 min at room temperature.

#### 2.1.4. Cell Count Analysis

For each APC, cell count analysis was conducted directly after centrifugation and before clotting. The white blood count, differential blood count, red blood count, and platelet count were automatically determined within 30–90 min after blood withdrawal on an ADVIA 120 Hematology System (Siemens, Erlangen, Germany) in the central laboratory of the University Medical Center Mainz for PRF, PRP and PFP as previously described [17]. Results were compared with EDTA full blood samples of the respective donors.

### 2.2. Macrophage Assays

#### 2.2.1. Isolation and Polarization of Human Monocyte-Derived Macrophages

The experiments’ macrophages were isolated from buffy coats provided by healthy donors and polarized as previously described [18]. This was performed in agreement with the Declaration of Helsinki, and the Ethics Committee of the Landesärztekammer Rhineland-Palatine approved the protocol (no. 837.019.10 (7028), approved 4 March 2010). Macrophages were harvested from petri dishes with Accutase (Thermo Fisher Scientific, Waltham, MA, USA, #00-4555-56) after 7 days in culture. Cells were plated at 1 million cells/well in six well-plates and polarized to “M1-like”, “M2-like”, or “M0” macrophages. “M0” macrophages were treated either with 1.5 mL of solidified PRP or PFP as well as PRF. All concentrates were prepared from the same donor. Following 2 days of incubation, cells underwent analysis via flow cytometry to determine alterations in the surface marker expression.

#### 2.2.2. Flow Cytometry

Solidified clots from the different concentrates were pressed through a 40 μm cell strainer (VWR International, Radnor, PA, USA, #732-2757) to create single cell suspensions and washed with prechilled PBS as previously described [18]. Macrophages were harvested from plates using Accutase (Thermo Fisher Scientific, Waltham, MA, USA, #00-4555-56). Cells were combined and washed once with PBS (Thermo Fisher Scientific, Waltham, MA, USA, #14190-094) prior to staining. Cells were stained for viability (Invitrogen, Carlsbad, CA, USA, #65-0866-14) and then underwent surface staining with the following antibodies as previously described: anti-CD40 (Immunotools, Friesoythe, Germany, #21270403), anti-CD163 (Life Technologies, Carlsbad, CA, USA, #A15792), anti-CD36 (Miltenyi Biotec, Bergisch Gladbach, Germany, #130-095-480), anti-CD206 (Miltenyi Biotec, Bergisch Gladbach, Germany, #130-100-152), anti-MSA4A (R&D Systems, Minneapolis, MN, USA, FAB7797R), anti-PD-L2 (Miltenyi Biotec, Bergisch Gladbach, Germany, #130-105-829), anti-CD14 (Invitrogen, Carlsbad, CA, USA, #48-0149-42), and anti-MHCII (HLA-DR) (BioLegend, San Diego, CA, USA, #307650) [18]. Samples were measured on a BD LSRII flow cytometer (BD Biosciences, San Jose, CA, USA). Data were analyzed using Cytobank software (www.cytobank.org), with doublets, debris, and dead cells excluded from the analysis [19]. The representing gating strategy is presented in Supplemental Materials Figure S2.

### 2.3. Proteomic Analysis

#### 2.3.1. Protein Extraction

The protein extraction procedure for the PRF (*n* = 3), PRP (*n* = 3), and PFP (*n* = 3) was carried out by employing our in-house protocol [20,21,22]. First, all the samples were centrifuged at 10,000× *g* for 10 min at 4 °C to separate the samples into two fractions of supernatant and pellet. The supernatant fraction of these samples (designated as sPRF, sPRP, and sPFP), which are composed of secreted proteins, were directly subjected to protein concentration estimation. However, the pellet fraction of these samples (designated as pPRF, pPRP, and pPFP) was homogenized using a T-PER Tissue Protein Extraction Reagent (Thermo Scientific Inc., Waltham, MA, USA) and stainless-steel beads in a Bullet Blender homogenizer (BBY24M Bullet Blender Storm, Next Advance Inc., Averill Park, NY, USA). The supernatant of the tissue homogenates was subjected to a buffer exchange and sample cleaning using 3 kDa centrifugal cut-off filters (Amicon Ultra 0.5 mL, Merck Millipore, Carrigtwohill, Ireland). Protein concentration estimation of all the samples was determined employing a bicinchoninic acid (BCA) protein assay kit (Pierce, Rockford, IL, USA).

#### 2.3.2. One-Dimensional Gel Electrophoresis (1DE) SDS-PAGE

Triplicates of samples in each group were subjected to 1DE (10 µg/lane) and separated under reducing conditions on 10-well precast 4–12% Bis-Tris mini gels with 1× NuPAGE MES SDS Running Buffer (both from Thermo Fisher Scientific, Rockford, IL, USA). Gels were run for 45 min at 4 °C at a constant voltage of 200 V. A pre-stained protein standard, SeeBlue Plus 2 (Thermo Fisher Scientific, Rockford, IL, USA), was used as molecular mass marker and a Novex Colloidal Blue Staining Kit (Invitrogen, Karlsruhe, Germany) was used to stain the gels according to the manufacturer’s instructions. Subsequently, gels were destained overnight to eliminate background staining and scanned on an Epson Perfection V600 Photo Scanner (Seiko Epson Corporation, Suwa, Nagano, Japan) [20,21,22].

#### 2.3.3. Mass Spectrometry (MS)-Based Proteome Analysis

The samples were subjected to in-solution trypsin digestion and peptide purification with SOLAµ™ SPE HRP plates (Thermo Fisher Scientific, Rockford, IL, USA) according to the manufacturer’s instructions. The resulting peptide eluate was concentrated to dryness in a centrifugal vacuum evaporator and dissolved in 0.1% formic acid with the final protein concentration of 50 ng/µL. The nano-liquid chromatography (nLC)-MS system employed comprised an EASY-nLC 1200 system (Thermo Scientific, Rockford, IL, USA) with an Acclaim PepMap RSLC, 75 µm × 25 cm, nanoViper analytical column (Thermo Scientific, Rockford, IL, USA) directly coupled to ESI-LTQ-Orbitrap-XL MS (Thermo Scientific, Bremen, Germany), as described elsewhere [22]. Two µL of each sample (100 ng) were used to fractionate peptides at a flow of 300 µL/min. Solvent A was LC-MS grade water with 0.1% (*v*/*v*) formic acid, and solvent B consisted of LC-MS grade acetonitrile with 20% (*v*/*v*) water and 0.1% (*v*/*v*) formic acid. The run of the resulting gradient per sample added up to a total time of 120 min. 0–90 min: 5–30% B, 90–100 min: 30–100% B, 100–120 min: 100% B. Briefly, an LTQ-Orbitrap was operated in a data-dependent mode of acquisition and survey full scan MS spectra from *m*/*z* 300 to 2000 were acquired in the Orbitrap with a resolution of 30,000 at *m*/*z* 400 and a target automatic gain control (AGC) setting of 1.0 × 10^6^ ions. The 10 most intense precursor ions were sequentially isolated for fragmentation and recorded in the LTQ.

#### 2.3.4. Label-Free Quantitative Proteomic Analysis

The acquired continuum MS spectra were analyzed utilizing MaxQuant (version 2.4.10.0) software [23,24,25,26,27]. The tandem MS spectra were searched against the *Homo sapiens* database (Uniprot, reviewed (Swiss-Prot); Accession date, 20 February 2024; Annotated proteins, 20433) with a target-decoy-based false discovery rate (FDR) for peptide and protein identification was set to 0.01. The generated protein list from the MaxQuant analysis was used for subsequent statistical analysis with Perseus (version 2.0.11) software [28]. First, a log_2_ transformation of all protein intensities was performed and the results were filtered to include only proteins with 100% valid measured values in at least one of the study groups. Missing values were subsequently imputated from a normal distribution in standard settings (width: 0.2, down shift: 1.8), enabling statistical analysis [27].

### 2.4. In Vivo Analysis

#### YOLK SAC Membrane (YSM) Assay

**Figure 1:**
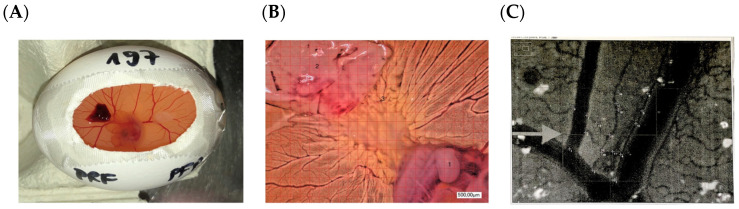
Yolk sac membrane (YSM) assay. (**A**): In ovo analysis of PRF (2) and PFP (*) in safe distance from the embryo (1). (**B**): Microscopical analysis of the induced vessels (3) in direct contact with the PRF (2) and in safe distance from the embryo (1). (**C**): Vessel analysis after grayscale conversion and background extraction with FIJI software (Version 2.9.0). Arrow: vessel.

A yolk sac membrane (YSM) assay was applied as previously described [1]. In brief, 8–10 mL of egg white of fertilized white Leghorn chicken eggs (LSL Rhein-Main, Dieburg, Germany) was removed after incubation at 38 °C at a constant humidity until the fourth day of embryological development. A 3 × 3 cm^2^ window was trimmed into the eggshell under sterile conditions and, after another 24 h, 5 × 5 mm of the cut PRF, PRP, and PFP clot, respectively, were inserted into the YSM (Figure 1). After 24 h, neo-vessel formation and branching points were evaluated using a digital microscope (KEYENCE, Neu-Isenburg, Germany) and the respective software (KEYENCE, Neu-Isenburg, Germany). After overlaying a grid (with a 500 μm side length) over the micrographs, ImageJ software (version 1.54, ACTREC, Navi Mumbai, India) was used for further analysis. At first, images (magnification 50×) were converted to grayscale and a correction of the background was conducted through image subtraction. Vessels were extracted from the background using automatic thresholding and all the vessels and branching points of the vessels in a defined region of interest per mm^2^ were analyzed. Afterwards, the embryos were euthanized by cutting the main vessels.

### 2.5. Statistical Analysis

All results were evaluated in mean values with their standard errors and illustrated as bar charts with error bars. Differences between all groups were analyzed with a Kruskal–Wallis rank sum test. After checking on normal distribution with a Shapiro–Wilk test, a Student’s *t*-test (in case of normally distributed values) or a Mann–Whitney test (for non-normal distributions) was used to check for statistical significances. For the statistical evaluation of the polarization experiments, data were analyzed by two-way ANOVA, corrected for multiple comparisons using Tukey tests. For statistical evaluation of the proteomic analysis, multiple sample tests (ANOVA, *p* ˂ 0.05) were used, followed by a Student’s two-sided *t*-test for pairwise comparisons between groups, with *p* ˂ 0.05 to identify the significantly differentially abundant proteins. The list of the significantly differentially abundant proteins in the designated comparison groups was used for functional annotation analysis employing an Ingenuity Pathway Analysis (IPA) [27]. The top enriched terms of the gene ontology cellular component (GOCC), molecular types, biological functions, upstream regulators, and canonical pathways of the differentially abundant proteins were presented with a *p*-value calculated using a Benjamini–Hochberg (B–H) multiple testing correction (one-sided Fisher’s exact test, -log B–H *p*-value > 1.3). Unsupervised hierarchical clustering analysis of the top canonical pathways and biological functions as well as the annotated differentially abundant proteins were performed according to a Euclidean distance (Linkage, average; Preprocess with k-means, enabled; Number of clusters, 300; Maximal number of iterations, 10; Number of restarts, 1) utilizing the Perseus software (version 2.0.11).

## 3. Results

### 3.1. First- and Second-Generation Platelet Concentrates Demonstrate Differences in Cell Content

**Figure 2:**
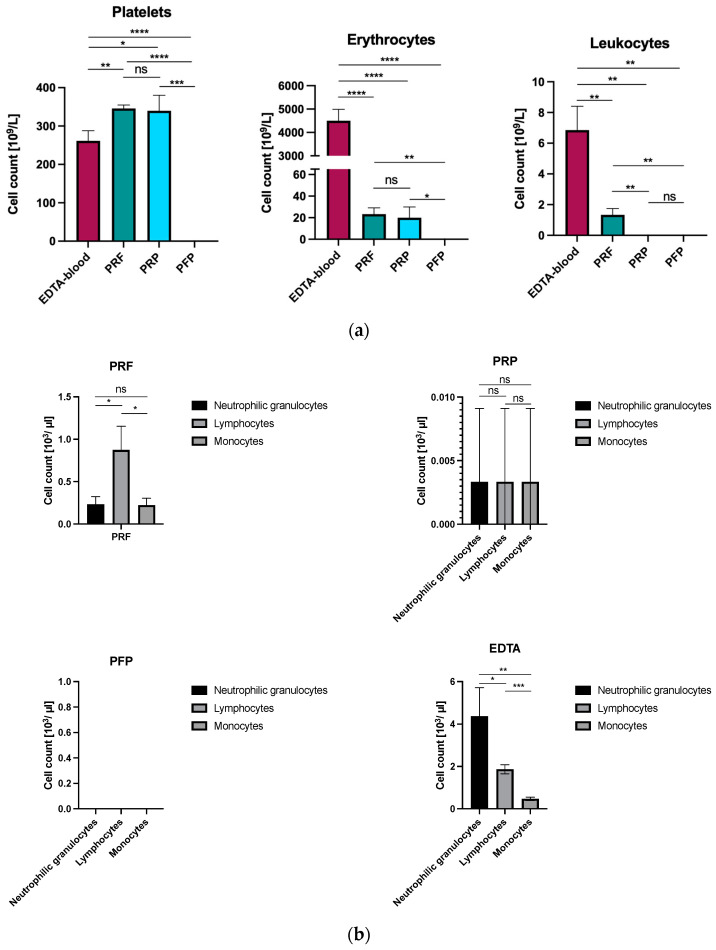
(**a**) Cytological analysis of PRF, PRP, and PFP compared to EDTA-blood in platelets, erythrocytes, and leukocytes (ns: *p* > 0.05; *: *p* < 0.05; **: *p* < 0.01; ***: *p* < 0.001; ****: *p* < 0.0001). (**b**) Cytological analysis of PRF, PRP, and PFP compared to EDTA-blood in neutrophilic granulocytes, lymphocytes, and monocytes (ns: *p* > 0.05; *: *p* < 0.05; **: *p* < 0.01; ***: *p* < 0.001).

Cytological analysis revealed differences in the platelet count, as well as red and white blood cell counts in EDTA-full blood compared to the PRF, PRP, and PFP of three healthy volunteers (Figure 2). For platelets, PRF and PRP revealed significantly (*p*-value PRF: 0.0065 and *p*-value PRP: 0.00484) higher amounts than full blood whereas PFP displayed no platelets at all.

Full blood showed significantly more erythrocytes and leukocytes compared to PRF and PRP. Further, no leukocytes were displayed in PFP and PRP whereas PRF contained significantly higher amounts of white blood cells compared to the other APCs (*p*-value PRF vs. PRP: 0.005) (Table 1, Figure 2a). In the differential blood count, neutrophils and lymphocytes as well as monocytes were considerably lower in PRP vs. PRF and full blood (Figure 2b).

### 3.2. First- and Second-Generation Autologous Platelet Concentrate as Well as Platelet-Free Plasma Polarize M0-Macrophages into a More “M0/M2-like” Phenotype

In previous studies, PRF showed the capacity to polarize macrophages towards a “M0/M2-like” phenotype which can be found in wound healing (Supplementary Materials: Figure S1) [18]. To assess that capacity for the other two APC PRP and PFP, M0 macrophages were treated and analyzed for altered surface marker expression via flow cytometry, adopting the same protocol which was used for PRF samples mentioned above [18].

**Figure 3:**
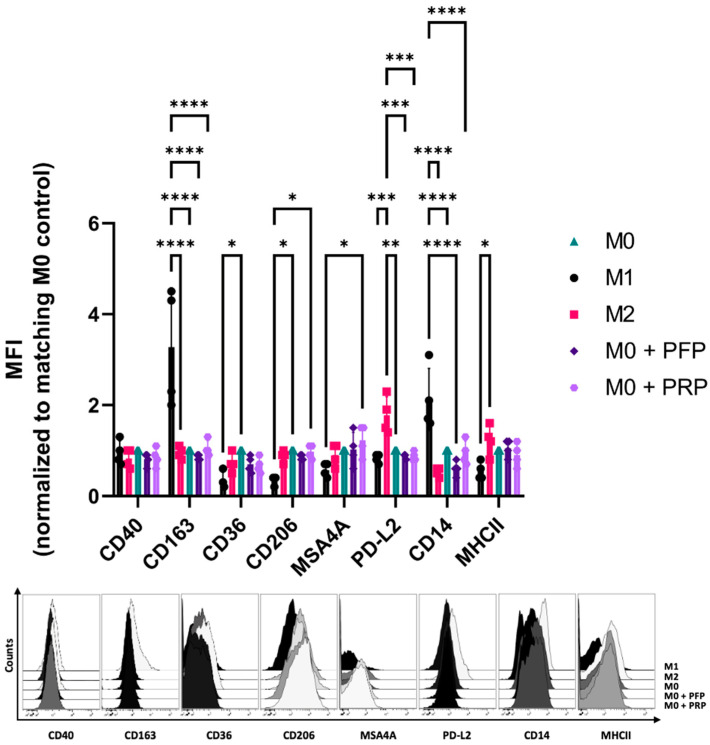
Platelet-rich plasma (PRP) and platelet-free plasma (PFP) polarize human monocyte derived macrophages to a “M0/M2-like” phenotype. Macrophages were polarized or treated with PRP or PFP. Following 2 days of incubation, surface marker expression was evaluated with flow cytometry. The bar diagram indicates the mean fluorescence intensity (MFI) of each marker, normalized to the matching donor “M0” control. The histograms show one representative result (*n* = 4 donors, means ± SD, * *p* < 0.05, ** *p* < 0.01, *** *p* < 0.001, and **** *p* < 0.0001 analyzed by two-way ANOVA, corrected for multiple comparisons using Tukey tests).

While PRP showed an increasing expression of M2 surface markers like CD206 (*p* < 0.05) and MSA4A (*p* < 0.05), the expression of M1 surface markers such as CD14 (*p* < 0.0001) and CD163 (*p* < 0.0001) was correspondingly decreased. PFP on the other hand, as the control, demonstrated no statistically significant increase in M2 surface markers, while also decreasing the expression of M1 surface markers like CD14 (*p* < 0.0001) and CD163 (*p* < 0.0001). In conclusion, both APC PRP and PFP, like PRF, demonstrated a shift to an M0/M2-like anti-inflammatory phenotype without showing any proinflammatory potential. When directly comparing PRP and PFP, we did not find a statistical significance that one APC is more effective than the other (Figure 3).

### 3.3. Proteome and Secretome Map of the APC Sub-Classes

**Figure 4:**
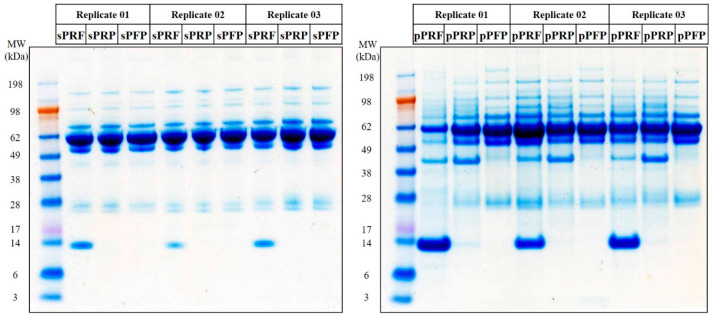
Representative protein profiles of the designated groups comprising the soluble proteins from sPRF, sPRP, and sPFP, and cell-derived proteins from pPRF, pPRP, and pPFP resolved in 1DE-PAGE after colloidal blue staining.

Protein profiles of the designated groups resolved in 1DE-PAGE comprising the soluble proteins (sPRF, sPRP, and sPFP) and derived collectively from different cell types (pPRF, pPRP, and pPFP) are as shown in Figure 4. A higher number of protein bands (at molecular weight > 50 kDa) were observed in the samples derived from cells (pPRF, pPRP, and pPFP) compared to sPRF, sPRP, and sPFP. Notably, a protein band with the molecular weight ~14 kDa, largely represented by human hemoglobin subunit beta (HBB) and hemoglobin subunit alpha-1 (HBA1) was only observed in the PRF samples compared to PRP and PFP (Figure 4).

**Figure 5:**
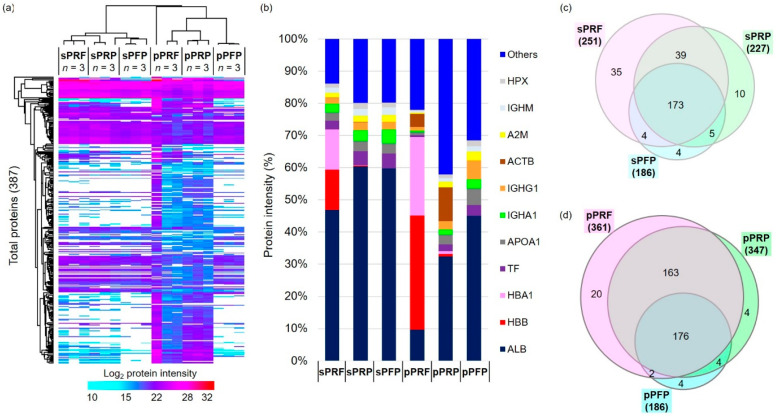
Label-free quantitative proteomics analysis of the supernatant and pellet fragments of the APC. (**a**) Heat map depicts the hierarchical clustering of 387 total proteins based on the log_2_ protein intensity corresponding to the designated groups. (**b**) Bar charts show the degree of mean percentage of top abundant proteins in the designated groups. (**c**,**d**) Venn diagrams illustrate the total number of proteins identified in all samples.

Label-free proteomic analysis identified a total of 387 proteins with a FDR of 1% from all three biological replicates of the respective designated groups (Figure 5a, complete protein list in Supplemental Table S1). The top 10 most abundant proteins identified were composed of approximately 60–85% of the total sum of all identified proteins, namely serum albumin (ALB), HBB, HBA1, serotransferrin (TF), apolipoprotein A1 (APOA1), Ig alpha-1 chain C region (IGHA1), beta-actin (ACTB, and alpha-2-macroglobulin (A2M) (Figure 5b). Furthermore, HBB and HBA1 were predominantly identified in the sPRF (~20%) and pPRF (~60%). The highest number of proteins was identified in the pPRF (361) and pPRP (347), while the lowest number of proteins was identified in the sPFP (186) and pPFP (186) (Figure 5c,d). Approximately 45% of the identified proteins were found in all the sample types. However, a higher number of overlapping proteins, 42%, were identified in the pPRF and pPRP compared to 10% in the sPRF and sPRP.

**Figure 6:**
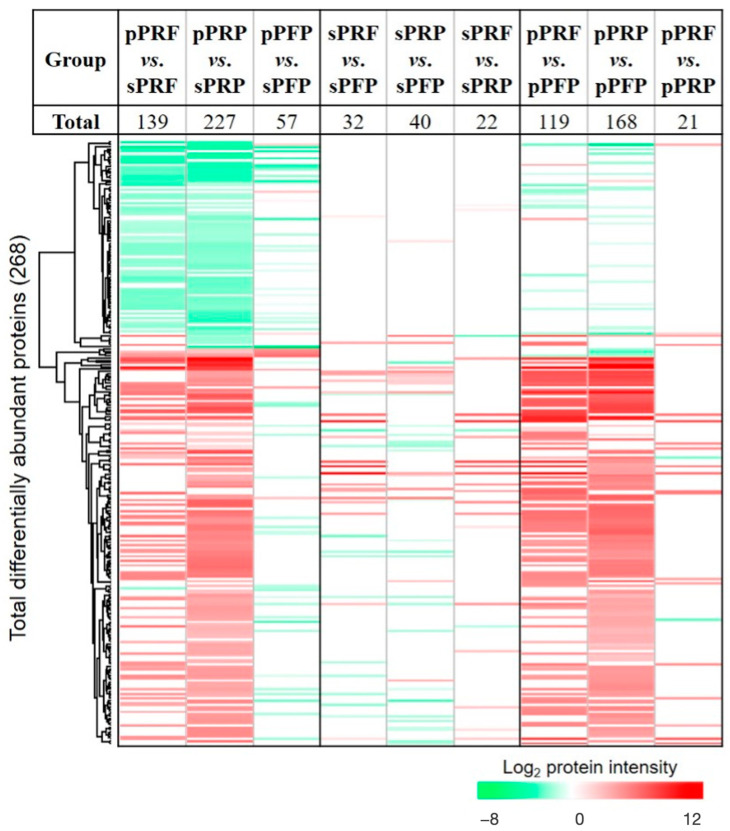
Heat map depicts the hierarchical clustering of the total of 268 significantly differentially abundant proteins in the designated comparison groups. Significance threshold is at *p* < 0.05. The up-regulated proteins are shown in red and the down-regulated proteins are in green.

Statistical analysis identified as many as 268 proteins to be significantly differentially abundant in the designated groups (Figure 6; Supplemental Tables S2 and S3). A large preponderance of these differentially abundant proteins was observed in the pPRF vs. sPRF (227), and pPRP vs. the sPRP (139) compared to pPFP vs. sPFP (57) (Figure 6). This observation showed a significant increment of the specific cluster of membrane proteins, namely alpha-actinin-1 (ACTN1), myosin-9 (MYH9), fructose-bisphosphate aldolase A (ALDOA), vinculin (VCL), and filamin-A (FLNA) yielded from the fractions homogenized with detergent from the PRP and PRF compared to PFP (Supplemental Table S2). The total number of significantly differentially abundant proteins in the soluble fractions of the sPRF vs. sPFP and sPRP vs. sPFP were 32 and 40, respectively (Figure 6).

**Figure 7:**
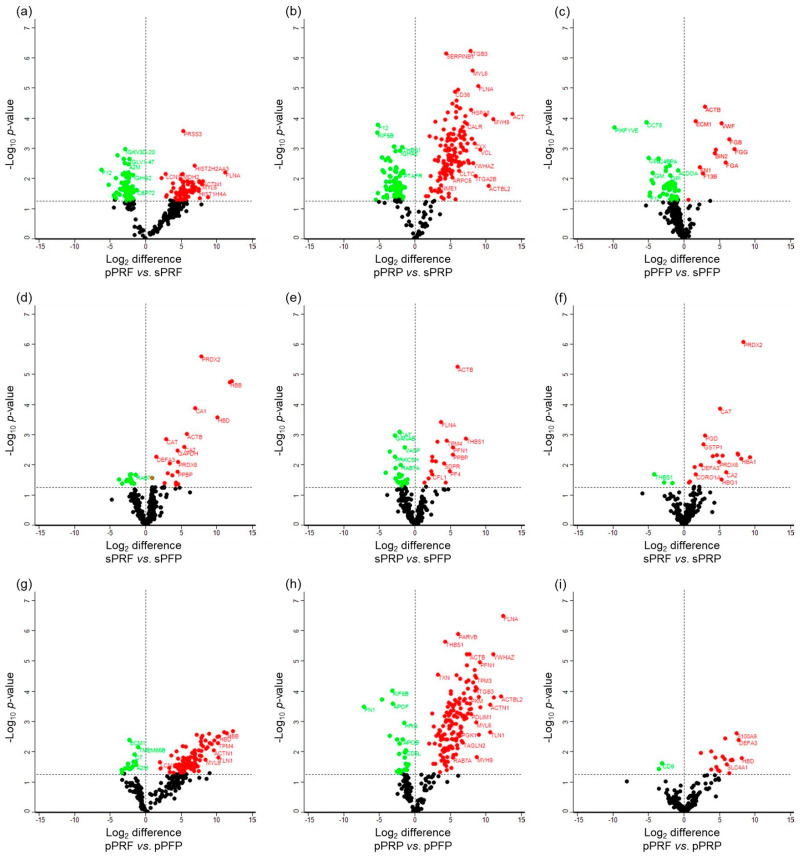
Volcano plots illustrate the significantly differentially abundant proteins identified based on the log_2_ difference in the (**a**) pPRF vs. sPRF, (**b**) pPRP vs. sPRP (**c**) pPFP vs. sPFP, (**d**) sPRF vs. sPFP, (**e**) sPRP vs. sPFP, (**f**) sPRF vs. sPRP, (**g**) pPRF vs. pPFP, (**h**) pPRP vs. pPFP, and (**i**) pPRF vs. pPRP. The significance threshold is at *p* < 0.05. The up-regulated proteins are shown in red and the down-regulated proteins are in green.

When comparing the groups of pPRF and pPRP, they collectively share 339 proteins that can be found in both APCs (Figure 5). In comparison to that, sPRF and sPRP only share 212 proteins but the total number of expressed proteins is also smaller compared to the pellet fraction. pPRF and pPRP displayed distinct proteomic profiles, with a particularly notable set of 21 proteins showing significant differential abundance, namely, protein S100-A9 (S100A9), histone H2A (HIST2H2AA3), neutrophil defensin 3 (DEFA3), annexin A3 (ANXA3), extracellular matrix protein 1 (ECM1), ankyrin-1 (ANK1), neutrophil gelatinase-associated lipocalin (LCN2), hemoglobin subunit delta (HBD), vimentin (VIM), HBB, carbonic anhydrase 1 (CA1), hemoglobin subunit alpha (HBA1), CD9 antigen (CD9), band 3 anion transport protein (SLC4A1), annexin A1 (ANXA1), leukocyte elastase inhibitor (SERPINB1), fibronectin (FN1), protein S100-A8 (S100A8), thrombospondin-1 (THBS1), histone H4 (HIST1H4A), and hemoglobin subunit gamma-1 (HBG1) (Figure 6 and Figure 7i). In comparison, the soluble fractions of PRF and PRP (sPRF vs. sPRP) also exhibited 22 differentially abundant proteins, among them, for example, proteins like peroxiredoxin-2 (PRDX2), catalase (CAT), 6-phosphogluconate dehydrogenase, decarboxylating (PGD), glutathione S-transferase P (GSTP1), alpha-actinin-1 (ACTN1), putative Ras-related protein Rab-1C (Rab-1B), peroxiredoxin-6 (PRDX6), and glutathione S-transferase omega-1 (GSTO1). The full comprehensive analysis of all differentially abundant proteins can be found in Supplements Table S2.

**Figure 8:**
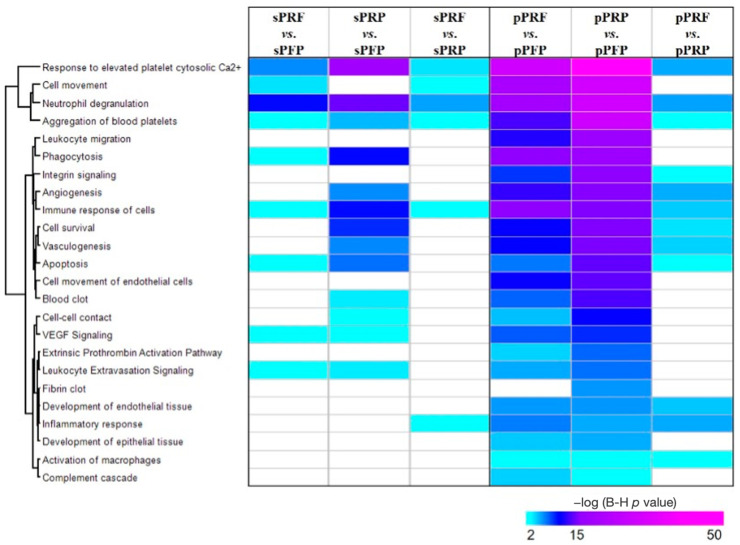
Heat map depicts the top significant enriched biological functions and canonical pathways of the differentially abundant proteins in the designated comparison groups. The significance of enrichment (−log_10_ (B-H *p*-value) is scaled by color intensity; *p* < 0.05.

Next, to decipher the functionalities of the differentially abundant proteins in PRF and PRP compared to PFP, an IPA tool [27] was employed to analyze the most significant canonical pathways and biological functions. The top significantly annotated pathways/biological functions associated with the pPRP vs. pPFP comprised a response to elevated platelet cytosolic Ca^2+^ (*p* = 5.3 × 10^−48^), cell movement (*p* = 4.3 × 10^−30^), neutrophil degranulation (*p* = 2.0 × 10^−29^), aggregation of blood platelets (*p* = 5.4 × 10^−29^), leukocyte migration (*p* = 2.5 × 10^−17^), and phagocytosis (*p* = 3.0 × 10^−17^) (Figure 8). Similarly, the aforementioned pathways/biological functions were also annotated to the pPRF vs. pPFP but with a lesser degree of significance compared to the pPRP vs. pPFP.

### 3.4. Second-Generation Autologous Platelet Concentrates Demonstrate Increased Pro-Angiogenic Potential

**Figure 9:**
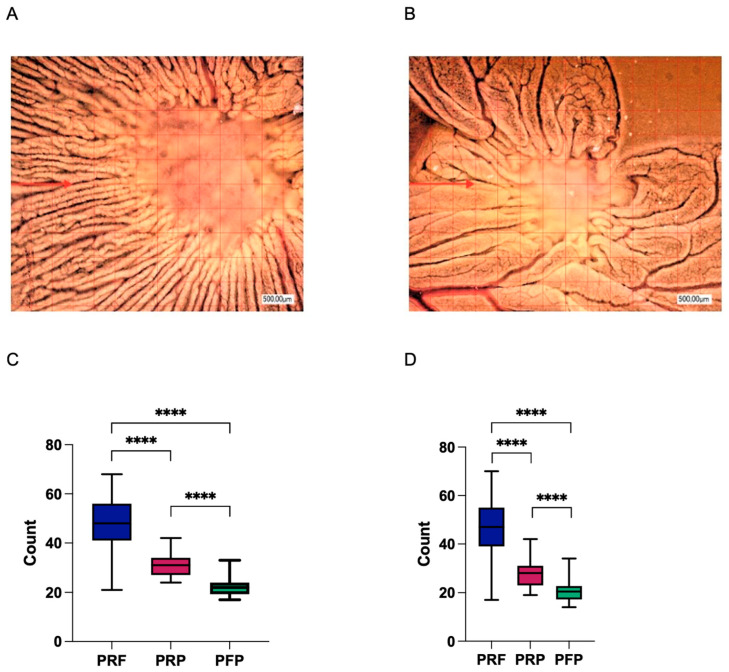
Microscopic analysis of vessel formation in the yolk sac membrane (YSM) assay for PRF (**A**) and PFP (**B**). The red arrow in (**A**) highlights the enhanced vessel formation in the PRF group compared to PFP. Panel (**C**) quantifies vessel formation, and (**D**) depicts the number of branching points, demonstrating a statistically significant increase in pro-angiogenic activity in the PRF group (**** *p* < 0.0001).

The pro-angiogenic characteristics of the respective APCs were analyzed by a YSM assay in vivo. Macroscopically, vessel formation was enhanced in the PRF group vs. PRP and PFP (Figure 9). PRF showed significant increased neo-vessel formation in comparison to PRP and PFP (each *p* < 0.0001). Furthermore, branching points were significantly increased in the PRF group vs. the PRP and PRF group (each *p* < 0.0001).

## 4. Discussion

This study analyzed the cell content, immunological potential, proteome, and secretome as well as the pro-angiogenic potential of first- and second-generation autologous platelet concentrates in comparison to a platelet-free plasma concentrate.

The results support the previously described differences in cell content between the first- and second-generation APCs in comparison to the platelet-free plasma. Of note, platelet numbers were similar between PRF and PRP. Furthermore, PRP and PFP showed similar immune capacities with the induction of the more M2-like polarization of macrophages as was already shown for PRF [18]. The label-free proteomics analysis demonstrated the comprehensive overview of the proteome landscape across different fractions derived from PRF, PRP, and PFP, highlighting significant differences in protein composition and abundance. Lastly, in vivo analysis demonstrated a significant increased neo-vessel and branching points formation for the second-generation concentrate PRF compared to PRP and PFP.

In the literature, many differences between first- and second-generation APCs are described, especially the centrifugation process that differs with the addition or omission of an anticoagulant substance in a one- or two-time procedure. As a result, both formulas vary in their cell composition. Both formulas had similar platelet counts, but PRF exhibited a higher red blood cell (RBC) content compared to PRP. This can be explained by the harvest technique that PRF is taken after the first and only centrifugation process. From other studies, it is known that platelets and leukocytes are concentrated in an intermediate layer located between RBCs and the fibrin clot. It is indispensable to preserve a small RBC layer at the PRF clot end to collect as many platelets and leukocytes as possible [29]. However, ideally the formulation of these biologics should contain minimal to no RBCs. Hypothetically, when APCs are delivered outside the blood stream to a local tissue microenvironment, the RBCs can undergo eryptosis that ultimately leads to secondary inflammatory conditions [30].

Overall, the leukocyte content was lower in PRP versus PRF, as well as neutrophils, lymphocytes, and monocytes. It is stated that white blood cells are indispensable for tissue repair [31], but their functions within APCs are still highly debated. Whereas several authors have proposed that including white blood cells may improve scaffold stability and antibacterial capabilities, others found contrary results. Additionally, a pro-inflammatory reaction due to the metalloproteases, pro-inflammatory proteases, and acid hydrolases generated by leukocytes must be estimated [31,32]. In our analysis, no differences were found concerning the immunogenic potential of both APCs in macrophages. Other studies show additionally similar antimicrobial properties between the formulas [32]. Surprisingly, PFP induced an M2 polarization of the macrophage to the same content as PRP. This can be partly explained by the finding from Szpaderska et al. that induction of thrombocytopenia in mice leads to altered wound healing but with no significant differences in the rate of re-epithelialization, collagen synthesis, and angiogenesis between thrombocytopenic and control mice [31].

To the best of our knowledge, this is the first study that comprehensively and comparatively analyzed the proteome and secretome of the different generations of APCs. Among the few studies that investigated the potential differences of the APC proteome, Al-Sharabi found biological similarities between the PRF secretome and human bone marrow stem cells with the top enriched proteins related to immune response, coagulation, wound healing, and platelet function [13]. Another study by Hermida-Nogueira et al. depicted the secretome of PRF at different time points. They found more than 700 proteins in total and could demonstrate that the secretome profile varies over time (days 3, 7, and 21) [12]. One of the major limitations of these studies is that the authors did not analyze the clot but only the secretome [12,13]. In addition, inter-individual differences can have an impact when analyzing these data. Bearing this in mind, Yaprak et al. found significant differences in the secretome between the respective samples reducing the commonly identified proteins of all samples to 35 differentially abundant proteins [33]. Our study investigated both variables to find differences in the proteome and secretome that can explain the described difference in the pro-angiogenic potential.

The proteome and secretome analysis revealed striking differences between the concentrates. The observation of a higher number of protein bands in cell-derived samples (pPRF, pPRP, and pPFP) compared to their soluble counterparts underscores the complexity of the proteome associated with cellular components. This is particularly evident in the significant differences in protein abundance between the cell-derived and soluble fractions, with a notable increase in specific membrane proteins in the cell-derived fractions such as ACTN1 and MYH9, for example. The identification of hemoglobin subunits (HBB and HBA1) predominantly in PRF samples further highlights the unique protein composition of these samples, potentially reflecting their distinct biological properties and preparation methods. The identification of specific protein clusters and the insights gained from pathway analysis highlight the complex biological functions associated with these samples. These findings not only enhance our understanding of the molecular basis of the therapeutic properties of PRF and PRP, but also open avenues for further research into their clinical applications. The significantly differential abundance of proteins, particularly in comparisons between pPRF vs. sPRF and pPRP vs. sPRP, reveals critical insights into the protein composition of these fractions. pPRF and sPRF exhibit a more complex protein network (261 and 251 proteins) compared to pPRP and sPRP (347 and 227 proteins), particularly within the pellet fraction, which may translate to enhanced therapeutic efficacy. Proteomic analysis revealed a significant upregulation of pro-angiogenic proteins such as FN1 or THBS1 in PRF compared to PRP. FN1, for instance, is a major extracellular matrix (ECM) protein involved in a wide range of physiological processes, including cell migration and wound healing [34], while THBS1 is involved in the regulation of several processes during tissue repair and angiogenesis [35]. These findings suggest that PRF may have superior therapeutic implications in wound-healing applications compared to PRP, aligning with the observed enhanced pro-angiogenic potential in vivo.

Although the increased abundance of angiogenic proteins may enhance wound healing and tissue regeneration, it also carries the potential risk of promoting oncogenesis [34]. A cell’s ability to induce angiogenesis to meet its excess supply of oxygen and nutrients, potentially facilitating their growth and metastasis, is the most significant hallmark of cancer [36]. For instance, FN1 has been shown to be involved in cancer metastasis [34], while THBS1 regulates angiogenesis and tumor perfusion [37]. It is also important to consider that the increased abundance of angiogenic proteins may carry heightened risks of tumor promotion in populations that have a predisposition toward inflammatory responses such as metabolic syndrome, for example, which is a condition driven by inflammation [38,39]. These potential risks underscore the necessity and importance of personalized treatment options to weigh benefits against specific risks and highlight the need for further studies examining APC safety and efficacy in these high-risk populations. However, currently there is only a limited number of studies investigating their impact on malignant cells, while parallel to that, there are also studies investigating a tumor-suppressive impact of APCs [40]. Although our study focuses on the regenerative and pro-angiogenic properties of APCs, further research is needed to investigate this topic to avoid potential high-risk situations, especially in patients with a history of cancer or those at high risk of malignancy, for example, patients with familial cancer.

The identification of a specific cluster of membrane proteins, including ACTN1, MYH9, and others, in the cellular fractions from PRP and PRF compared to PFP, suggests a distinct cellular or structural component associated with these samples. These proteins are known to play crucial roles in cellular structure, motility, and signaling, indicating their potential involvement in the biological functions of PRP and PRF. The significant pathways and biological functions identified, particularly in the comparison of pPRP vs. pPFP, such as a response to an elevated platelet cytosolic Ca^2+^, cell movement, and neutrophil degranulation, underscore the potential roles of these proteins in mediating specific cellular responses and functions.

A fundamental difference between both generations of APCs is the stable fibrin network that evolves in the second-generation derivates such as PRF [41]. Within this fibrin net, cells, their released growth factors, cytokines such as VEGF, and many more are “trapped”. This leads to a slow and steady release of these molecules [42]. Kobayashi et al. summarize that “PRP can be recommended for fast delivery of growth factors whereas PRF is better suited for long-term release” [9]. Whereas the total amount of released growth factors was previously described higher in PRF vs. PRP [8], others observed no statistical difference [43]. We found a higher vasoformative response for the first- vs. second-generation APCs in the YSM assay. In a similar approach, Kobayashi et al. found no significant differences for the total vessel number comparing PRP and PRF. However, they describe a higher potency for PRF vs. PRP. The relatively low number of eggs (*n* = 5) in their CAM analysis may further explain the data [43]. Of note, the authors describe the same low pro-angiogenic potential of PPP (platelet-poor plasma) compared to the PFP used here. As a possible explanation, TGF-β and PDGF that are concentrated in both PRP and PRF preparations, directly stimulate the proliferation of fibroblasts and collagen production [43].

There are several limitations to this study. It only investigated a small sample size of healthy donors with no history of smoking. This could lead to limitations in the generalizability of findings to a broader population because of sampling bias. It would be interesting for future research to analyze the APCs of a larger population, considering their health and possible risk factors to represent the landscape of patients in a clinical setting. The mass spectrometry-based proteomics approach, while powerful, may not always achieve the sensitivity levels of ELISA or microarrays for detecting very low-abundance proteins without extensive sample preparation and enrichment steps. The pro-angiogenic and chemokines protein markers (e.g., VEGF, PDGF, IL-6, IL-8, CCL2) often require highly sensitive detection methods. Therefore, they were not detected in this proteomics study.

Furthermore, the process of manufacturing, especially PRP and PFP, can lead to differences, for example, in the cell count due to the multi-step centrifugation and pipetting process. This study tried to minimize that risk and standardize this process by reducing the number to one investigator carrying out the manufacturing process. In addition, this study was conducted in a controlled laboratory setting, possibly limiting its validity in a clinical setting. Therefore, future research should aim to validate these findings in patient-related contexts by clinical comparative studies.

## 5. Conclusions

The analyzed APCs exhibit both an anti-inflammatory effect in vitro but also demonstrate significant differences in cell content, proteome, and secretome that resulted in an altered vasoformative reaction in vivo. Here, a second-generation APC displayed a higher vessel number and branching points than the first-generation concentrate. The distinct proteome profiles of PRF and PRP suggest the potential for targeted therapeutic applications, leveraging the unique properties of these proteins for regenerative medicine, wound healing, and other clinical applications. From these results, the inclusion of leukocytes into the formula did not result in a pro-inflammatory response in vitro. Furthermore, the dense fibrin architecture with the growth factors and respective proteins encapsulated at the regeneration site led to an enhanced pro-angiogenic effect. Therefore, a second-generation concentrate seems superior to optimize wound healing. These data can help in the clinical translation and support the versatile clinical applications in regenerative medicine for second-generation autologous platelet concentrates. This should be further evaluated in clinical comparative studies.

## Figures and Tables

**Table 1:** Summary table of cell counts in different autologous platelet concentrates (PRF, PRP, PFP) and full blood.

Parameter	Full Blood (EDTA)	PRF	PRP	PFP	Statistical Significance (*p*-Value)
Platelet Count (×10^9^/L)	261.3 ± 12.5	345.7 ± 20.3	369.7 ± 15.2	0	*p* < 0.01
Erythrocyte Count (×10^9^/L)	4503 ± 300	23.33 ± 1.5	20.00 ± 1.3	0	*p* < 0.05
Leukocyte Count (×10^9^/L)	6.853 ± 0.4	1.333 ± 0.2	0.003 ± 0.001	0	*p* < 0.001

## Data Availability

The original contributions presented in the study are included in the article/Supplementary Material; further inquiries can be directed to the corresponding author.

## Supplementary Materials

The following supporting information can be downloaded at: https://www.mdpi.com/article/10.3390/bioengineering11111171/s1, Figure S1: Liquid platelet rich fibrin (iPRF) polarizes human monocyte derived macrophages to a “M0/M2-like” phenotype; Figure S2: Example flow cytometric gating strategy for macrophages. Doublets, debris, and dead cells were excluded from analysis; Table S1: Supplemental table S1_PRF vs. PRP; Table S2: Supplemental table S2_PRF vs. PRP; Table S3: Supplemental table S3_PRF vs. PRP.
