# Supplementary material for: First- vs. Second-Generation Autologous Platelet Concentrates and Their Implications for Wound Healing: Differences in Proteome and Secretome
Source: Bioengineering (Basel). 2024 Nov 20;11(11):1171. doi: 10.3390/bioengineering11111171 (PMC11591784; doi:10.3390/bioengineering11111171)
Supplement: Supplementary file 1 [file bioengineering-11-01171-s001.zip › Supplemental Figures.pdf]

Supplemental Material

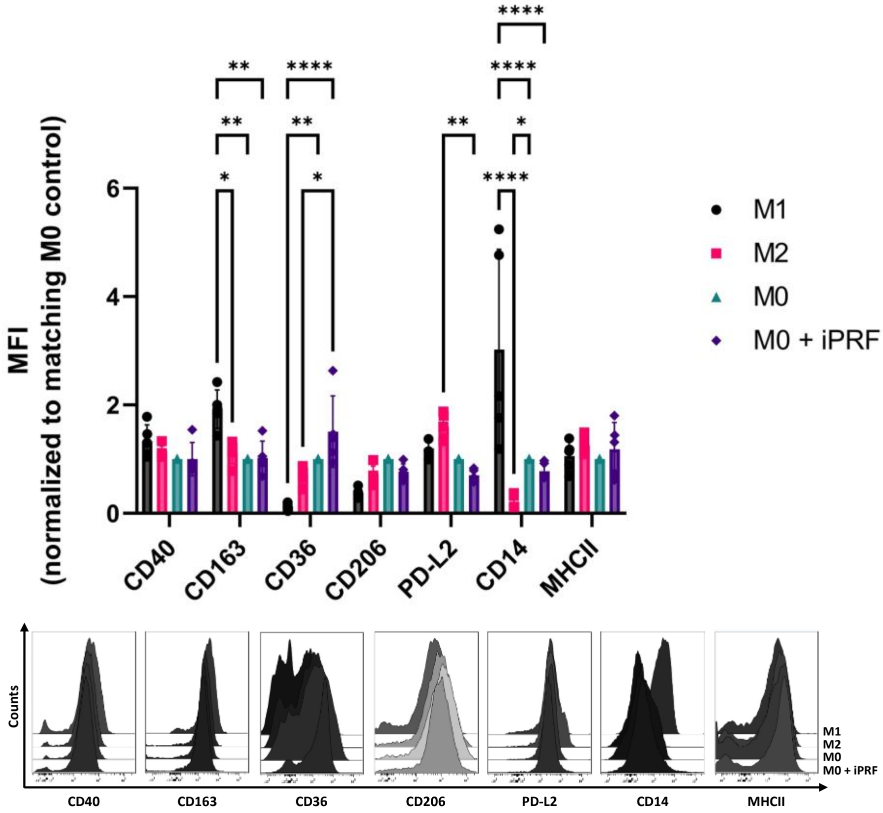

Supplemental figure S1: Liquid platelet rich fibrin (iPRF) polarizes human monocyte derived macrophages to a "M0/M2-like" phenotype. Data modified with permission from Trzeciak et al., 2022, Figure 4 [18]. Macrophages were polarized or treated with iPRF. Following 2 days of incubation, surface marker expression was evaluated with flow cytometry. Bar diagram indicates the mean fluorescence intensity (MFI) of each marker, normalized to the matching donor "M0" control. The histograms show one representative result of three independent experiments (n=5 donors, means ± SD, * $p < 0.05$, ** $p < 0.01$, *** $p < 0.001$, and **** $p < 0.0001$ analyzed by two-way ANOVA).

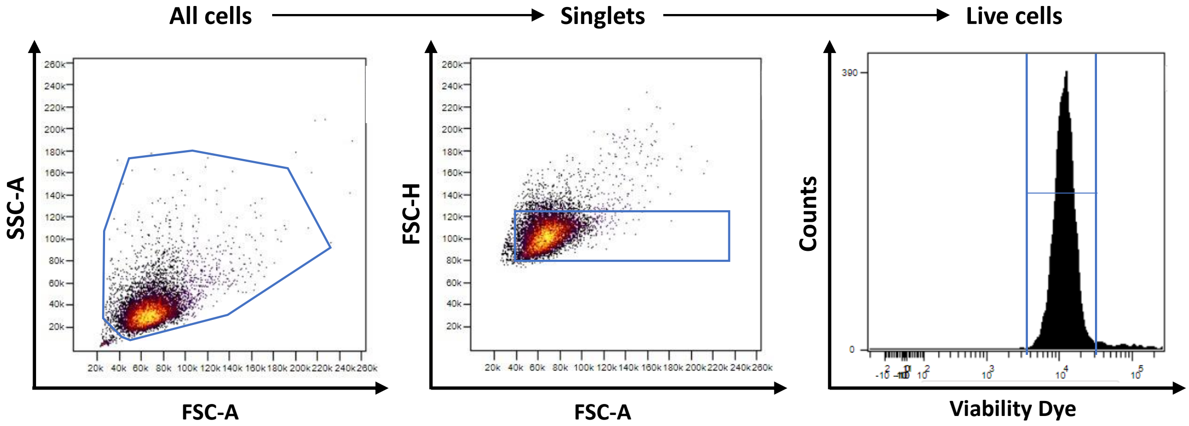

Supplemental figure S2: Example flow cytometric gating strategy for macrophages. Doublets, debris, and dead cells were excluded from analysis.
